# Implementing a high-efficiency similarity analysis approach for firmware code

**DOI:** 10.1371/journal.pone.0245098

**Published:** 2021-01-12

**Authors:** Yisen Wang, Ruimin Wang, Jing Jing, Huanwei Wang

**Affiliations:** 1 State Key Laboratory of Mathematical Engineering and Advanced Computing, Zhengzhou, China; 2 Information Engineering University of China, ZhengZhou, China; Northeastern University at Qinhuangdao, CHINA

## Abstract

The rapid expansion of the open-source community has shortened the software development cycle, but the spread of vulnerabilities has been accelerated, especially in the field of the Internet of Things. In recent years, the frequency of attacks against connected devices is increasing exponentially; thus, the vulnerabilities are more serious in nature. The state-of-the-art firmware security inspection technologies, such as methods based on machine learning and graph theory, find similar applications depending on the known vulnerabilities but cannot do anything without detailed information about the vulnerabilities. Moreover, model training, which is necessary for the machine learning technologies, requires a significant amount of time and data, resulting in low efficiency and poor extensibility. Aiming at the above shortcomings, a high-efficiency similarity analysis approach for firmware code is proposed in this study. First, the function control flow features and data flow features are extracted from the functions of the firmware and of the vulnerabilities, and the features are used to calculate the SimHash of the functions. The mass storage and fast query capabilities of the SimHash are implemented by the pigeonhole principle. Second, the similarity function pairs are analyzed in detail within and among the basic blocks. Within the basic blocks, the symbolic execution is used to generate the basic block semantic information, and the constraint solver is used to determine the semantic equivalence. Among the basic blocks, the local control flow graphs are analyzed to obtain their similarity. Then, we implemented a prototype and present the evaluation. The evaluation results demonstrate that the proposed approach can implement large-scale firmware function similarity analysis. It can also get the location of the real-world firmware patch without vulnerability function information. Finally, we compare our method with existing methods. The comparison results demonstrate that our method is more efficient and accurate than the Gemini and StagedMethod. More than 90% of the firmware functions can be indexed within 0.1 s, while the search time of 100,000 firmware functions is less than 2 s.

## 1. Introduction

In recent years, the scale of the open-source community has expanded rapidly, promoting the capabilities of the software development industry. Developers can directly apply the code they need from the community, which shortens the software development cycle. However, the excessive use of open-source code not only brings convenience but also accelerates the spread of vulnerabilities. For example, device manufacturers often use open-source libraries without security inspection, and these libraries have a high probability of containing vulnerabilities [1]. Moreover, hackers can insert malicious code into the library, and then place the modified library in the open-source community libraries to attract downloading by unsuspecting users. When these libraries are downloaded and used by developers, the threat brought in by the vulnerability will spread to many products and affect many users [2]. Especially in the field of the Internet of Things (IoT), the number of interconnected embedded devices has increased exponentially in recent years. At the end of 2018, the number of interconnected devices -worldwide is approximately 7 billion, and the total number of connected devices is expected to reach 22 billion by 2025. Attacks on interconnected devices also show an increasing trend year by year for two main reasons: 1. The open-source public libraries used by device manufacturers contain many known and unknown vulnerabilities; 2. The architectures of firmware are diverse, which leads to one architecture’s vulnerability spreading to many other devices.

Detailed information about vulnerabilities cannot be obtained normally because, even if the manufacturer patches the vulnerability, they will not always announce the patch information. Therefore, analyzing vulnerabilities will cost analysts significant time and energy. Compared to the analysis of vulnerabilities of personal computer (PC) and mobile phone application programs, it is more difficult to analyze firmware vulnerabilities. Firmware analysis has the following difficulties: 1. Inability to obtain firmware source code [3, 4]; 2. Equipment peripherals are complex, which leads to the low success rate of firmware simulation [5, 6]; 3. Instruction set architectures (ISA) and operating systems of the firmware are diverse, and the compiler and compile optimization levels are not unified; moreover, many vendors will develop private file systems [7]; 4. The high overhead of traditional matching approaches prevents large-scale firmware security analysis [8]; 5. The details of vulnerability functions and firmware patches may be unobtainable.

The first difficulty is common in many other areas, and fortunately there has been much research on binary code analysis, such as code plagiarism detection [9], malicious code analysis [10], and software vulnerability discovery. The second difficulty is unique to the embedded security field, where there are many mature dynamic analysis tools for software, such as PANDA [11], WinDbg, among others. However, there is less research on firmware dynamic analysis. AVATAR [6] and AVATAR2 [12] are currently the most advanced platforms for firmware dynamic analysis; AVATAR runs the firmware alternately on physical devices and the QEMU emulator, and uses Selective Symbolic Execution (S2E) to perform symbol execution and taint analysis to explore security issues. However, this method is too expensive to apply on a large scale. Firmadyne [5] can realize the whole system simulation of firmware, but this tool can only simulate a few simple router devices. The third difficulty is also unique to the embedded security field; at present, there are many research efforts on binary analysis in a single architecture such as, for example, [13–15]. However, research on cross-architectures are few, although there are some representative researches [7, 8, 16–19]. Various publications [7, 8, 16, 17, 18] select a small number of control flow features to represent the function, which will lead to the loss of function information. Zuo et. al [19] uses long short-term memory to embed the basic blocks, but this has no uniform instruction embedding model. Difficulties 4 and 5 are the primary problems of existing technologies; the overhead of firmware cross-architecture analysis is high, including model training and retraining, generation of feature embedding, data preparation, among others, which leads to poor extensibility. Existing firmware vulnerability detection technologies need to extract features from known vulnerability functions and then detect similar firmware functions by feature matching. However, vulnerability functions and patch information cannot be obtained normally and such vulnerabilities cannot be detected by existing technologies.

Existing firmware vulnerability detection technologies play an important role in the field of IoT security, but these technologies share one or more the following shortcomings: incomplete features, high overhead, and poor extensibility. Because of the great numbers, critical locations and ease of attack of connected devices, a method that can quickly detect firmware security vulnerabilities and locate patches is urgently needed to realize rapid and frequent security inspections of devices.

This paper proposes a high-efficiency similarity analysis approach for firmware code. The method extracts the control flow features and data flow features of the firmware functions and assigns different weights to different features according to their importance. The weighted features are used to calculate the function’s SimHash, and the pigeonhole principle is used to realize the mass storage and fast query of the SimHash. And then the fine-grained similarity analysis at the basic block (BB) level is executed for similar function pairs. The method can not only implement large-scale firmware function security analysis, but can also locate the patches of patched firmware. Section 2 introduces the background of the method we use. Section 3 introduces the overview of our method. Sections 4 detail the implementation of our method. Section 5 evaluates our method, section 6 and section 7 discusses the related work, and section 8 concludes the study.

Our main contributions are as follows.

- *W*e propose a novel SimHash-based firmware function similarity analysis method, which can implement large-scale firmware function security analysis. We design a basic block-level similarity analysis method, which identifies the location of a firmware patch without the need for vulnerability function information.
- *W*e obtain the data flow features extracted from the data dependency graph and the features generated by Angr, which can improve the accuracy of function similarity. We design a ReliefF algorithm, which assigns weights to different features depending on their importance.
- *W*e implemented a prototype and present the evaluation results. The experimental results demonstrate that the efficiency of our method is higher than that of the StagedMethod and Gemini methods. More than 90% of the firmware functions can be retrieved within 0.1 s, while the search time of 100,000 firmware functions is less than 2 s. The proposed approach does not need model training, thus unknown firmware can be analyzed directly.

## 2. Background

### 2.1. Firmware code similarity analysis

Code similarity analysis is a common technique for malicious code analysis that can be used in firmware security analysis. However, firmware code similarity analysis is very different from the traditional PC code similarity analysis. Firstly, the firmware code is encapsulated in the EEPROM or FLASH chip of the device. In general, analysts cannot get the firmware source code. Therefore, the traditional open source similarity analysis method is not suitable for firmware code similarity analysis. Second, the instruction set architecture (ISA) of firmware is diverse, including X86, ARM, MIPS, PPC, and so on. The operating system, compiler and compiler optimization are also not identical, many vendors will also develop private file system. The traditional single-architecture code similarity analysis method is not suitable for firmware code similarity analysis. Third, both the number of firmware and the amount of firmware code are large. Some traditional code similarity analysis methods are time-consuming and not suitable for large-scale firmware code similarity analysis. Fourth, due to the complex peripherals of devices, firmware is difficult to realize dynamic simulation or hardware debugging. So, firmware code similarity analysis is a hard work.

The syntax of two firmware functions compiled from the same source code may be different, but the semantics are equivalent. To overcome the syntax differences of homologous functions, existing technologies select robust features to represent the firmware function, such as the control flow graph (CFG), function call flow graph (FCG), data flow graph and other semantic features. The CFG of the function represents all the paths traversed during the execution of the function, the basic block is the node of the CFG, and the relationship among basic blocks are the edges of the CFG. A basic block is a sequential execution of a series of instructions, with only one entrance and an exit. FCG represents a program’s functionality and the invocation relationships among functions. Programs compiled from same source code are semantically equivalent, regardless of processor architecture. The data dependence graph (DDG) of a function includes variables such as registers and constants and so forth. David et al. [20] uses strands to represent a program (a strand is a data flow slice of a basic block), to analyze the similarity among functions by comparing the number of identical strands.

To overcome the diversity of processor architectures, instructions from different architectures can be converted to an intermediate representation (IR) and then compared. Angr [21] is a classic program analysis tool that can convert instructions to Valgrind VEX-IR. Angr can also generate the function CFG, and then generate the control dependence graph and the DDG according to the CFG. The DDG allows one to determine what statements a given value depends on. Function data features can be obtained from DDG.

### 2.2. SimHash

A locally sensitive hash (LSH) maps the sample data from its original space to a new space, making the adjacent points in the original space remain adjacent with a high probability in the new space, while the non-adjacent points in the original space remain non-adjacent with a high probability in the new space. LSH is primarily used to search and find similar data in mass datasets. LSH is calculated from the sensitive hash function family, which can be expressed as (*r*1, *r*2, *p*1, *p*2), where *dist*: *F* × *F* → *R* is the distance measure function, *r*1 and *r*2 (*r*1 < *r*2) are the distances between any two eigenvectors calculated by *dist*, and *p*1 and *p*2 are two probabilities (*p*1 > *p*2). If for any *h* ∈ *H*;*x*, *y* ∈ *F* satisfies the following two conditions, then the hash function set *H* is sensitive:

$$\begin{array}{c} \begin{array}{c} If\;dist(x,y)<=r1,then\;Pr[h(x)=h(y)]>=p1; \end{array} \end{array}$$

$$\begin{array}{c} \begin{array}{c} If\;dist(x,y)>=r2,then\;Pr[h(x)=h(y)]<=p2; \end{array} \end{array}$$

SimHash is an LSH based on a random hyperplane; it constructs multiple random hyperplanes. The angle between the high-dimensional vector of the original data and the multiple random hyperplanes determines the similarity between the two vectors. The greater the similarity, the higher the probability that the two vectors are on the same side of the random hyperplane. SimHash is typically used for webpage de-duplication and is very fast. We use SimHash to compare the similarities between the two firmware functions.

### 2.3. Symbolic execution

Symbolic Execution is a common technique used in program analysis; it expresses variable values in the program as symbols without executing the program, and constraint solving is carried out by collecting constraint conditions. The technique can analyze the semantics of a program, or a part of the program, by adding tags. The basic idea of symbol execution is to convert the input value into the symbol value at the entrance of the program CFG, and convert the numerical operation of the original concrete value into the algebraic representation of the operation on the symbol. Symbolic execution gathers the branches of the program as a constraint item. The constraints solver then determines the range of input values for different paths of the program, and selects the appropriate input values during the test, so that the error detection module can find bugs or errors on different paths. We use symbolic execution and the constraints solver to determine the basic block’s semantic equivalence.

## 3.Overview

Code similarity analysis is an effective method to realize firmware security inspection. The analysis calculates the similarity between firmware functions and firmware vulnerability functions. The function with high similarity has a high probability of being a vulnerability function. We propose a high-efficiency similarity analysis approach for firmware code, which can realize large-scale firmware function similarity analysis to identify the firmware patch location without detailed information of the vulnerability.

**Fig 1:**
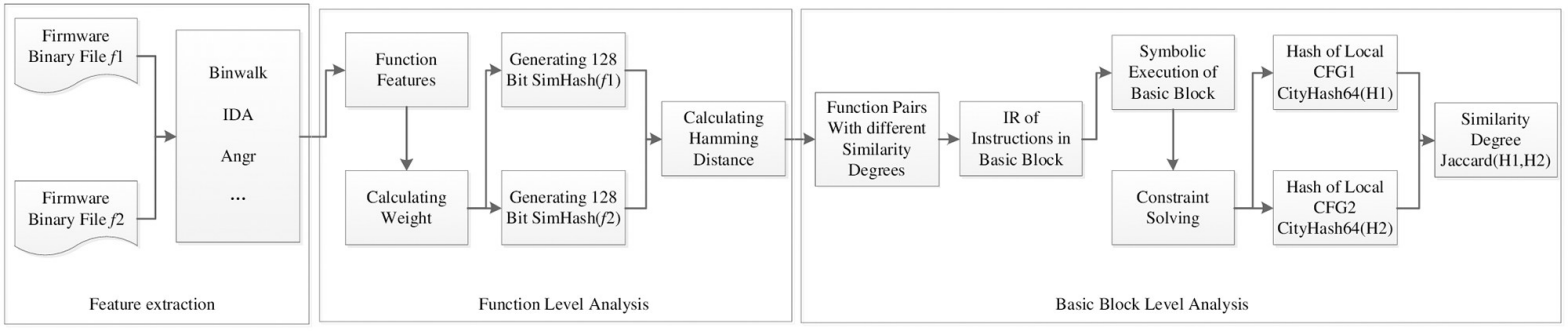
Approach overview.

The overview of our method is shown in Fig 1. The inputs are two firmware binaries to be compared. Firstly, Binwalk is used to perform a preliminary analysis of the firmware, to determine the basic information of the firmware processor architecture and operating system, and decompress the firmware to gain access to the firmware file system. For interesting binary files, the interactive disassembler (IDA) plug-in is used to extract the CFG features, and Angr is used to extract the DDG features. The 128-bit SimHash values of the different firmware functions are calculated according to the features and feature weights. Then, by calculating the Hamming distance between the SimHash of the different functions, the magnitude of the Hamming distance becomes a measure of the similarity among functions. To reveal patch locations without knowing the details of the vulnerability function, a basic block-level analysis is carried out on function pairs having similarity within a defined range.

The overview can be divided into three modules:

1. Feature extraction
  The richer the function features, the more accurately represented are the firmware functions. Wang et al. [17] extracted 50 control flow features which could represent the control flow property of a firmware function. Additionally, Angr was used to extract data features from the firmware function. The firmware function can be better represented by combining control features and data features. The ReliefF algorithm is used to calculate the weights of the features. Further details on firmware function feature extraction are provided in section 4.
2. SimHash similarity analysis
  The SimHash of the firmware function can be calculated by using the features and feature weights of the function. Hamming distance is the criterion for judging the similarity of the SimHash of different functions; the smaller the Hamming distance is, the greater the similarity. The number of functions in our firmware function database (DB) is large and growing rapidly, To realize the large-scale firmware function security inspection capability, the pigeonhole principle is used to provide the mass storage and fast query of the SimHash. Further details on the SimHash of firmware function similarity analysis are provided in section 5.
3. Basic block-level similarity analysis
  To realize the basic block analysis of the firmware function under different architectures, the function instructions should be converted into IR. Analyzing the function’s IR is equivalent to analyzing the function. The input and output of the basic blocks are expressed as symbols, and the constraint solver is used to determine whether the semantics of the two basic blocks are equivalent The basic block transfer paths are analyzed to calculate the similarity of the local control flow graphs (LCFGs) of different functions. Basic block-level analysis can determine the location of firmware patches. Further details on basic block similarity analysis are provided in section 6.

**Definition 1** *(Local Control Flow Graph) The local control flow graph, or LCFG in short, is part of the CFG*, *G*_*LCFG*_ = < *BB*, *BB*_*neigh*_, *E* >, *where BB is the target basic block*, *BB*_*neigh*_ *are the neighbors of the BB*, *E* ⊆ *BB* × *BB*_*neigh*_ *is a set of edges representing the connections between BB and BB*_*neigh*_s.

## 4.Design

### 4.1. Feature selection

In order to analyze the firmware function similarity, it is necessary to select the features that can represent the function. The quality of the features directly determines the accuracy of the function similarity analysis. Because of the variety of processor architectures and operating systems of the firmware, and the variety of compilers and compile optimizations used by the manufacturers, the similarity analysis of the firmware function needs to cross the architectures, operating systems and compilers, which increases the difficulty of the analysis. We select features that can overcome the heterogeneity of the processor architecture to analyze the similarity of the firmware functions. The control flow features and data flow features of the function are the functional properties of the function, and these features will not change with the change of processor and compiler. However, most of the existing technologies only select the control flow features, but ignore the data flow features, which causes the loss of function information.

We select both control flow features and data flow features to represent the firmware function, where the control flow features are selected from the CFG and function call flow graph, including: statistical features, structural features and invocation features. Statistical features typically include the number of instructions, proportion of different types of instructions, standard deviation, variance, mean, among others. The structural features include the structure of the graph, the number of basic blocks, the number of edges between basic blocks, the depth and width of the graph, among others. The invocation features include function calls and the number of calls. Wang et al. summarized a rich set of control flow features [17]. The data flow features are extracted from DDG, which are generated by Angr. The nodes of the DDG represent the number of data points in the function, including registers, variables, among others, while the edges of the DDG represent the data dependencies between nodes.

A total of 55 features were selected for function similarity analysis, as shown in Table 1. Different firmware function features play different roles in similarity analysis, and they have different degrees of importance. If all features are given the same weight, the similarity accuracy will be affected. Therefore, it is necessary to calculate and assign different weights to features according to their importance. The Relief (Relevant Features) algorithm [22] is a classic filtering feature selection method, which solves the problem of feature selection for binary classification. The ReliefF algorithm, which can solve multi-classification problem, is an improvement on the Relief algorithm. Weighting features according to the distinguishing ability of them in the same cluster instance and different cluster instances, the larger the weight is, the stronger the classification ability of the feature is; the smaller the weight is, the weaker the classification ability of the feature is. For m instances and n feature data sets, the time complexity of ReliefF is *O*(*mn*), which is suitable for the weight calculation of firmware features in this study.

**Table 1:** Function features.

Feature type	Feature name	Weight
Function call feature	No. of calls	0.02683
	No. of called	0.02779
	No. of indirect calls	0.0181
	No. of lib functions	0.02744
Instruction feature	No. of instructions	0.02105
	No. of arguments	0.0115
	No. of local variate	0.01063
	No. of Basic Block indegree	0.01355
	No. of Basic Block outdegree	0.01478
	No. of Arithmetic instructions	0.02701
	No. of Bit Manipulation instructions	0.01362
	No. of Data Transfer instructions	0.02610
	No. of String instructions	0.03174
	No. of Processor Control instructions	0.02887
	No. of Iteration Control instructions	0.02187
	No. of Interrupt instructions	0.02081
	No. of Load instructions	0.01311
	No. of Store instructions	0.01587
	No. of Call instructions	0.03079
	Strings	0.03158
	No. of strings	0.02815
	Constants	0.03934
	No. of constants	0.01876
Statistical feature	Entropy of instructions	0.00739
	Entropy of Arithmetic instructions	0.00681
	Entropy of Bit Manipulation instructions	0.00248
	Entropy of Data Transfer instructions	0.0045
	Entropy of Execution Transfer instructions	0.0052
	Entropy of String instructions	0.01099
	Entropy of Processor Control instructions	0.00458
	Entropy of Iteration Control instructions	0.00409
	Entropy of Interrupt instructions	0.0042
	Skewness of instructions	0.0242
	Kurtosis of instructions	0.02116
	Standard deviation of instructions	0.01227
	Mean of instructions	0.01029
	Variance of instructions	0.01169
	Z-score of instructions	0.01364
Structure feature	Stack	0.01652
	No. of CFG nodes	0.02573
	No. of CFG edges	0.01892
	No. of CFG paths	0.01884
	No. of nodes in shortest path	0.02185
	No. of loops	0.01235
	Base block depth	0.01402
	Base block average depth	0.0253
	Base block maximum depth	0.0276
	Base block average breadth	0.01297
	Base block maximum breadth	0.01383
	Density of graph	0.02375
Data flow feature	No. of DDG nodes	0.02108
	No. of DDG edges	0.02789
	No. of DDG paths	0.01391
	DDG average depth	0.01850
	DDG maximum depth	0.02137

OpenSSL was selected as the training set, and it was compiled with different compilers to obtain different architecture library files. The calculation process of 55 firmware function features can be represented by Algorithm 1.

**Algorithm 1** Function feature selection algorithm

**Input:** Iteration number *T*, function set *D*, nearest neighbor number *k*, Feature set *S*, Sampling number *m*

**Output:** Weight vector *W*

1: Initialize feature weight *W*[*S*] = 0

2: **for** *t* in *T* **do**

3:  Randomly select function *f*

4:   Similar neighbor set *Sim*_*f*_ = *H*_*j*_(*j* = 1, 2, …, *k*) and Dissimilar neighbor set *DisSim*_*f*_ = *M*_*j*_(*j* = 1, 2, …, *k*)

5:  **for** *s* in *S* **do**

6:   $W\left(s\right)=W\left(s\right)-\sum_{j=1}^{k}diff(s,R,Hj)/(mk)+\sum_{j=1}^{k}diff(s,R,Mj)/(mk)$

7:  **end for**

8: **end for**

9: **return** *W* = *W*(*S*)

In the algorithm, the input data set *D* is a function extracted from the binary files which were obtained from the *OpenSSL* software library and compiled with different compilers (*GCC*, *Clang*) and different optimization options (*O*1, *O*2, *O*3) under the *X*86, *ARM*, and *MIPS* architectures. Each function has 18 similar functions, and the dimension of the weight *W* is 55. In line 4, *k* similar neighbor functions are selected from similar functions, while non-similar neighbor functions are randomly selected from other functions, the function similarity is calculated by using Euclidean distance. In line 6, *diff*(*s*, *R*1, *R*2) is the difference between sample *R*1 and *R*2 on feature *s*, and the calculation formula is shown below. In line 9, after *T* iterations, the weight vector is finally returned.

$$\begin{array}{c} diff\left(s,R1,R2\right)=\frac{\left|R1[s]-R2[s]\right|}{max(s)-min(s)} \end{array}$$

### 4.2. SimHash function similarity

#### 4.2.1. SimHash similarity calculation

Compared to the traditional hash algorithms, LSH can reflect the degree of similarity of two texts. Data points in the original space are transformed and mapped to the new space. Data points that are close to each other in the original space are also likely to be close in the new space, while points that are distant in the original space are likely to be distant in the new space. A hash table is created by transforming all the data in the original content set, and the mapping of the original content set is distributed to various locations in the hash table. The original content is divided into many subsets using the method of hash function mapping, and the data in each subset has the characteristics of adjacency and of small quantity. Therefore, the problem of querying adjacent data in a very large set is simplified to the problem of querying adjacent data in a small set, and the processor utilization is significantly reduced. SimHash is a typical LSH designed for text similarity detection, and its principle can also be applied to function similarity detection.

**Fig 2:**
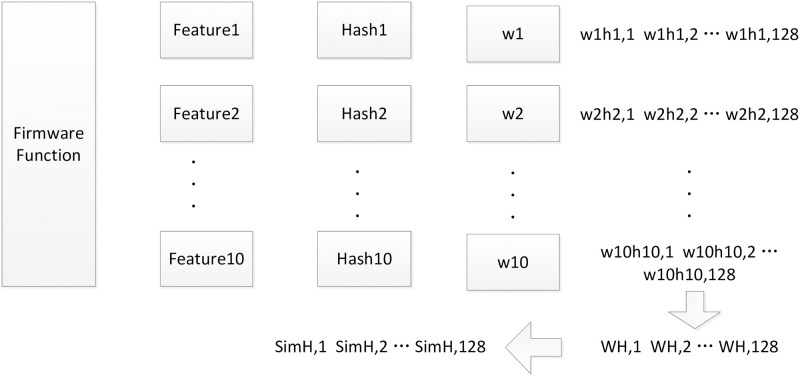
Principle of firmware function SimHash.

SimHash can be used to represent the firmware function. Functions with high similarity will be mapped closer together, while functions with low similarity will be mapped further apart. IDA is used to extract the features of the firmware function, and then calculate the SimHash of the function. The calculation principle of SimHash is shown in Fig 2, and the calculation process is as follows:

1. Extract firmware function features which are shown in the second column of Fig 2.
2. Calculate the feature hashes which are shown in the third column of Fig 2; the feature hash is a 128-bit signature composed of ‘0’ and ‘1’.
3. The ReliefF algorithm is used to calculate the weight of each feature to populate the fourth column.
4. Weights of the features are shown in the fifth column; the bit with a feature hash of 1 is multiplied by the positive weight, and a bit with a characteristic hash of 0 is multiplied by the negative weight. For example, the hash value of feature ′*Firmware*′ is “101100001010”, with the weight of *W*_1_ = 2. The weight feature is $\begin{array}{c} W\left(Firmware\right)=101100001010\cdot W_{1}=W_{1}\;-W_{1}\;\\ W_{1}\;W_{1}\;-W_{1}\;-W_{1}\;-W_{1}\;-W_{1}\;W_{1}\;-W_{1}\;W_{1}\;-W_{1}\\ =2-2\;2\;2-2-2-2-2\;2-22-2 \end{array}$.
5. The next step is to combine all the weighted features, and combine the sequences of all features into one sequence. For example, assuming the weight of ′*Similarity*′ is 7 -7 -7 7 7 7 -7 7 -7 7 -7 7 7, then the sum of feature ′*Firmware*′ and feature ′*Similarity*′ is 9 -9 -5 9 5 5 -9 5 -5 5 9 5.
6. The last step is to reduce the dimensionality of the resulting sequence. In the n-bit signature of the sequence, the bits greater than 0 are set to 1, and the bits less than 0 are set to 0. For example, the sequence 9 -9 -5 9 5 5 -9 5 -5 5 9 5 can be changed to 1 0 0 1 1 1 0 1 0 1 1 1. The reduced dimensionality data is the SimHash.

The Hamming distance is used to measure the similarity among firmware function SimHash, and is calculated as follows:

$$\begin{array}{c} SimBit\left(f_{1},f_{2}\right)=Hamming\left(SimHash\left(f_{1}\right),SimHash\left(f_{2}\right)\right) \end{array}$$ (1)

When SimHash is used for text comparison, a Hamming distance of 3 is an appropriate point. Two texts are considered similar when the Hamming distance less than 3, while the two texts are considered to be non-similar when the Hamming distance is greater than 3. However, functional similarity is different from text similarity; distance 3 is applicable to large functions with more basic blocks, but it is not applicable to small functions with fewer basic blocks. Experimental results show that if the distance 3 is used as the judgment boundary of similar functions, the false positive rate is relatively high. Many experiments have verified that a Hamming distance of 7 is an appropriate point for firmware functions; functions have a high degree of similarity if the Hamming distance between them is within 7. The formula for functional similarity is

$$\begin{array}{c} Sim(f1,f2)=SimBit(f1,f2)/128 \end{array}$$ (2)

#### 4.2.2. SimHash storage and quick retrieval

Firmware functions are stored in the firmware function DB in the form of 128-bit SimHash, while vulnerability functions are stored in the vulnerability function DB in the form of 128-bit SimHash. The firmware function DB and vulnerability function DB have two primary purposes: (1) When a new vulnerability function is available, it is possible to quickly detect the existence of similar vulnerability functions in the firmware function library; (2) When analyzing new firmware, the vulnerability function library can be used to detect whether there are similar vulnerability functions in the new firmware. However, as the amount of firmware collected increases, the firmware function DB will become very large, and the speed of the firmware vulnerability detection will decrease. Therefore, the storage and retrieval of firmware functions need to be optimized.

For the 128-bit firmware function SimHash, there are two approaches for searching for all signatures that have a Hamming distance less than 7: One is to look for all variation combinations of the 128-bit SimHash within a distance of 7; however the number of combinations is $C_{128}^{7}$, which about 90 billion queries. Another approach is to pre-generate all the various combinations of the 128-bit SimHash within distance 7, which requires an expansion of the original space more than 90 billion times. The first method is time consuming, while the second method is space consuming. To improve query efficiency and reduce storage space, the pigeonhole principle can be adopted.

**Fig 3:**
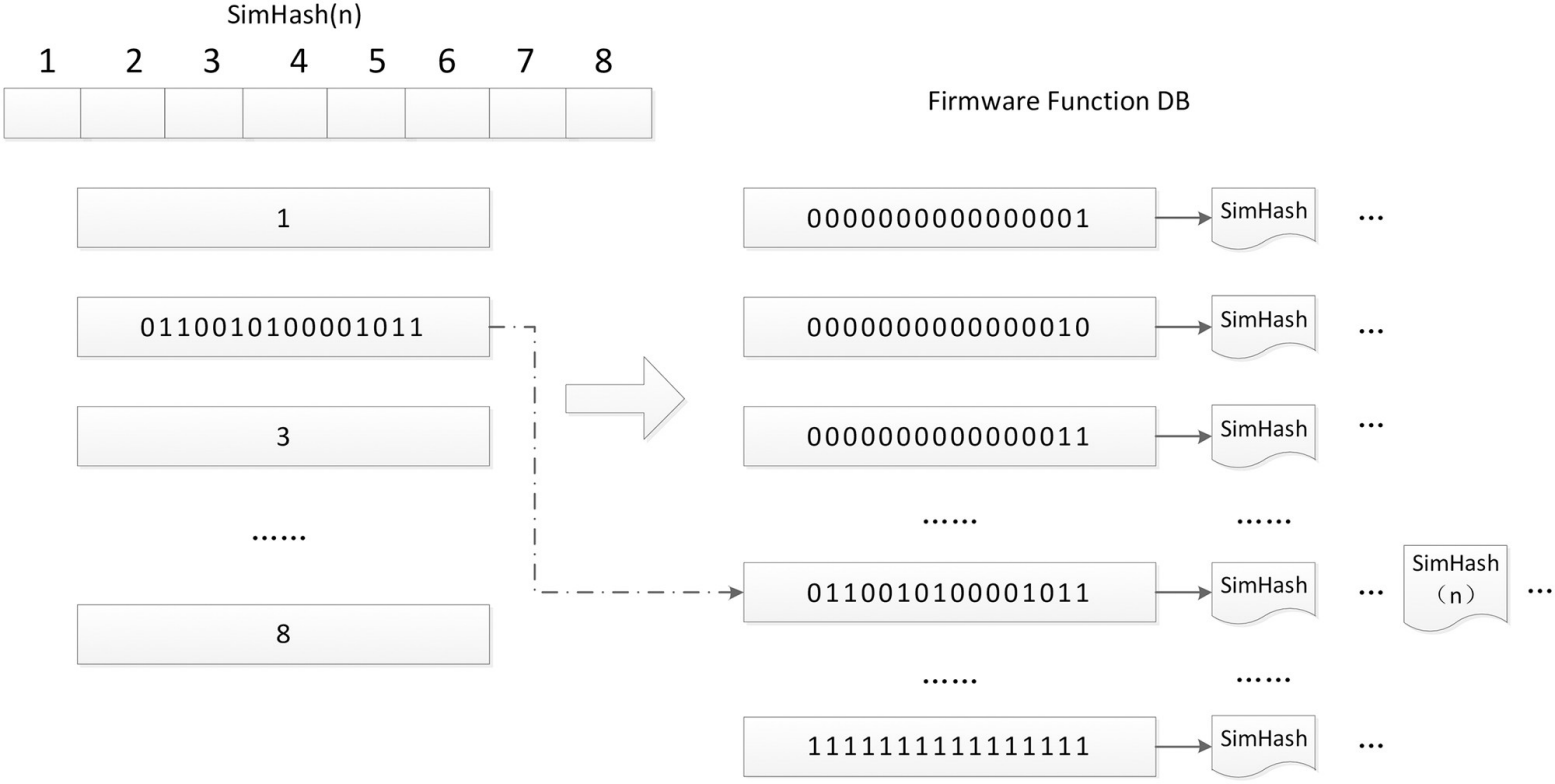
Storage of firmware function SimHash.

*4.2.2.1.Storage of the firmware function SimHash.* The storage of the firmware function SimHash is shown in Fig 3, Firstly, the 128-bit SimHash of the firmware function is divided into 8 pieces, each of which is 16-bit binary code. If the Hamming distance is within 7, at least one pieces of SimHash is identical; Each 16-bit binary code is searched in the database. If there is no element on the label of the corresponding position, the SimHash is directly added to the list. If there are elements on the label of the corresponding position, it is appended to the end of the list.

*4.2.2.2. Search of the firmware function SimHash.* The 128-bit SimHash is split into eight pieces, each of which is 16 bits binary code, and at least one of pieces of SimHash is identical to the label in the database. Each 16-bit binary is searched in the database to determine if there are elements on the label of the corresponding position. If there are elements, the SimHash is compared with all elements on the list.

The method can realize mass storage and fast query of firmware function. For example, if the sample database has about ten million firmware functions, then there are about 2^24^ SimHashes, so the search of each of the pieces of the SimHash will return 2^(24 − 16)^ results, that is 256 results, and a total of 8 * 256 = 2048 results are returned. If the sample database has one billion samples, 2 million results will be returned. Compared with the previous two methods, this method greatly improves the retrieval efficiency.

### 4.3. Basic block semantic similarity analysis

SimHash can be used to detect whether there are suspicious vulnerability functions similar to the vulnerability functions in the firmware. However, details of vulnerability functions are not available in many cases and vendors will not always publish patch information. Moreover, the patch code is usually very small, such as the change of boundary conditions and judgment conditions, among others. The features of CFG and DDG will not change in this case, and SimHash cannot determine the patch location, requiring fine-grained basic block-level analysis.

#### 4.3.1. Basic block semantic analysis

**Fig 4:**
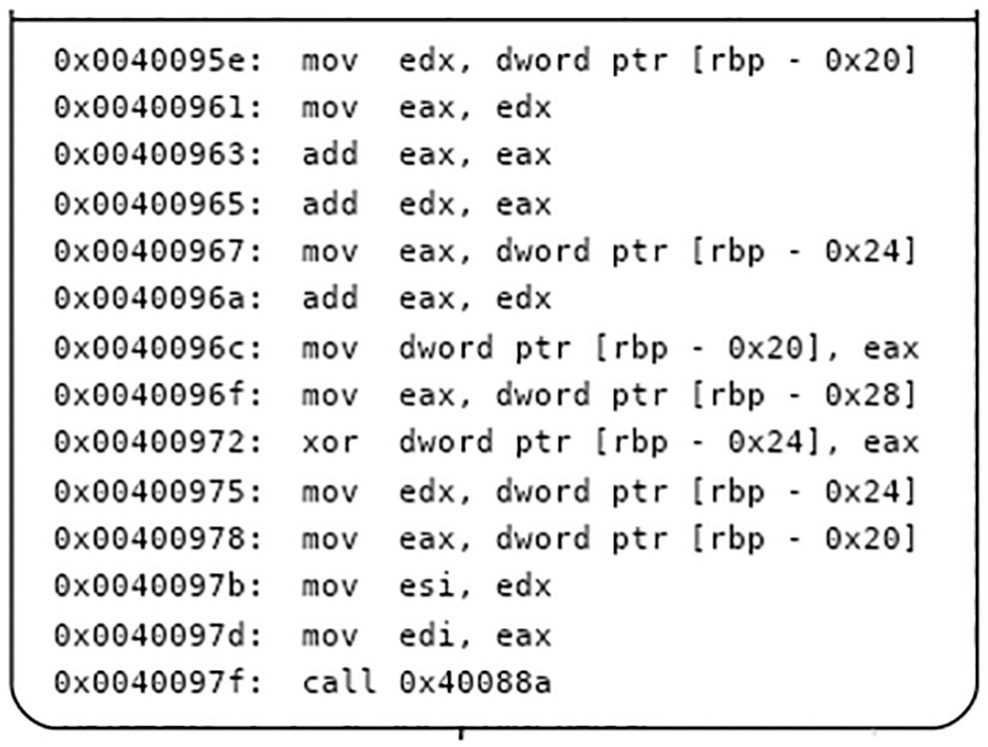
Basic block instruction.

**Fig 5:**
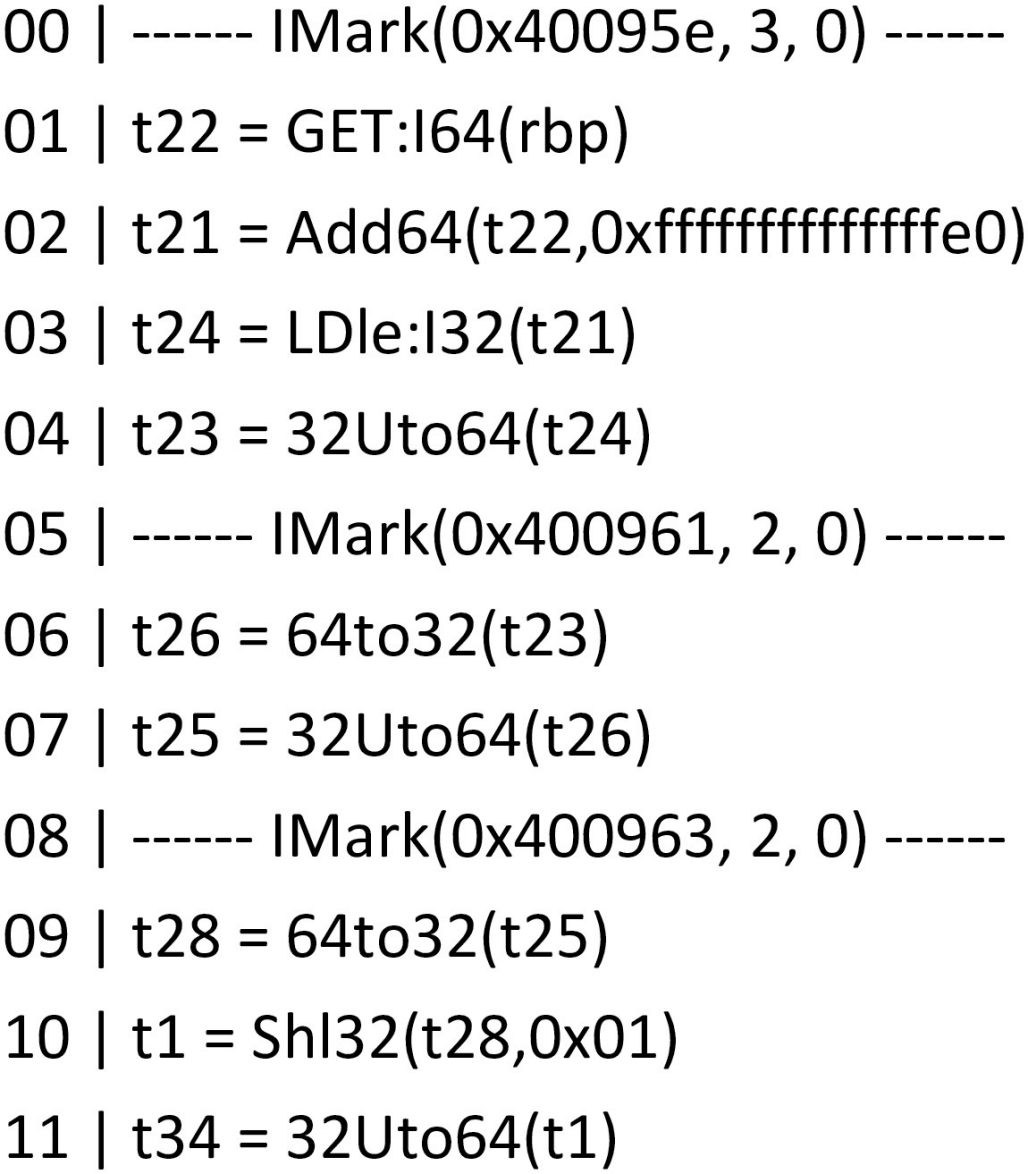
Basic block instruction IR.

**Fig 6:**
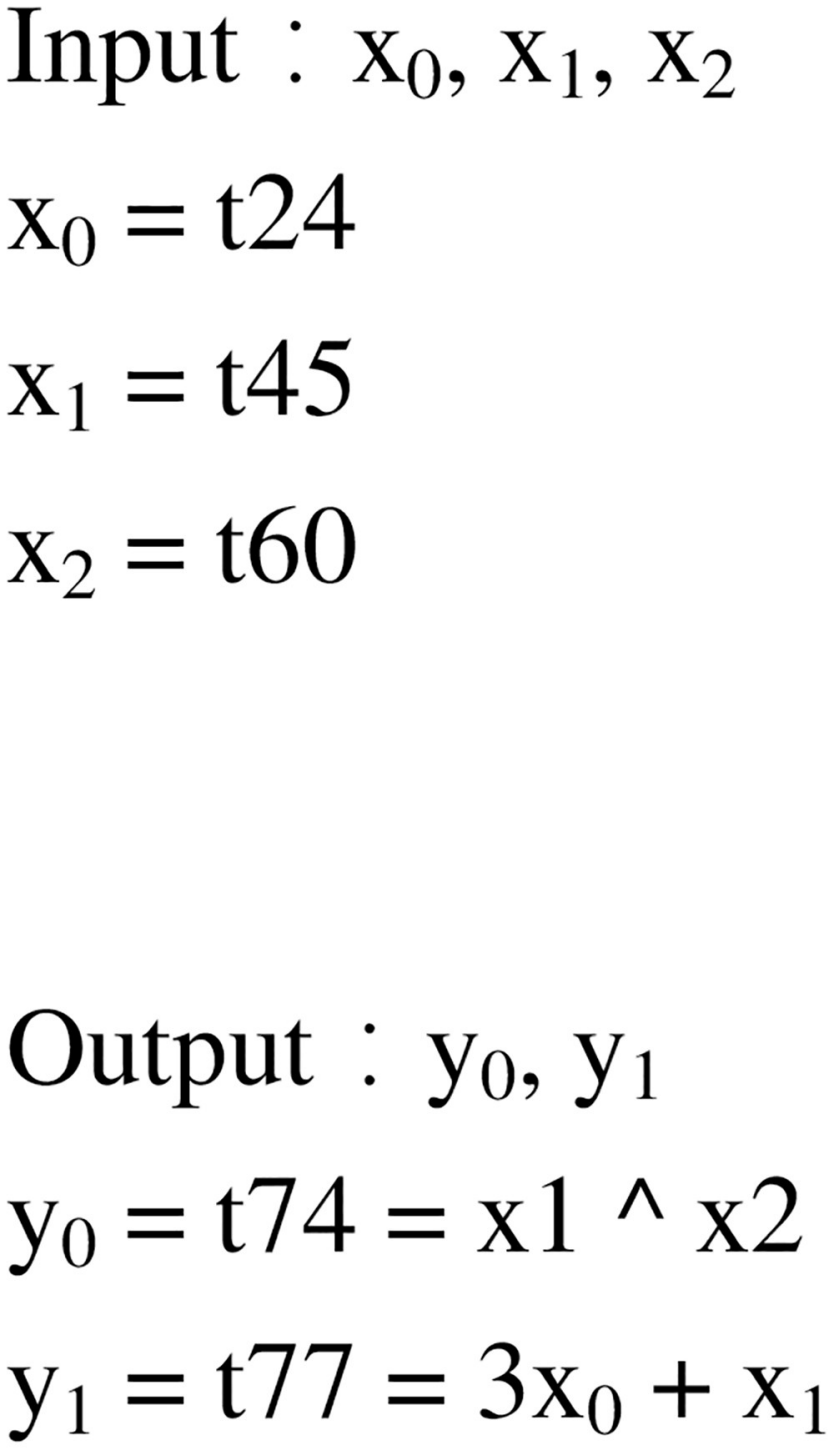
Input and output symbolic representation.

Using symbol execution to represent the input and output of basic blocks, and using the constraint solver to compare the output of basic blocks, it can be determined whether the two basic blocks have the same semantics. An instance is used to describe the process of basic block semantic analysis, as shown in the Figs 4, 5 and 6. Fig 4 is a screenshot of a basic block of the function CFG analyzed by Angr, with a total of 14 instructions. Fig 5 is the IR of the instructions in the basic block. There are 83 intermediate variables and 75 lines of code. Due to space limitation, only 11 lines of code are listed. Fig 6 is the symbolic representation of the inputs and outputs of the basic block.

The processor architectures for firmware are diverse, and the registers, variables, offsets and so on are also different. The IR represents the variables in the form of symbols, which can realize firmware cross-architecture analysis. There are three input variables in Fig 4, namely [*rbp* − 0*x*20], [*rbp* − 0*x*24], and [*rbp* − 0*x*28]. The IRs of [*rbp* − 0*x*20], [*rbp* − 0*x*24], and [*rbp* − 0*x*28] are *t*24, *t*45 and *t*60, respectively. Symbols are used to represent the input variables and the final outputs of the basic block. The output variables in Fig 4 are *esi* and *edi*, which are represented by the IR as *t*74 and *t*77, and the symbol representations in Fig 6 are *x*_1_∧*x*_2_ and 3*x*_0_+ *x*_1_. Symbol execution often faces challenges, such as path explosion and constraint solution, in practical applications, but our use of symbol execution in the basic block mitigates these problems.

When all variables in the basic block are represented by symbols, the constraint solver is invoked to determine whether the two basic blocks are semantically equivalent. It provides the same input for the two basic blocks, which may use different registers and variables, thus it is difficult to determine the assignment of the input symbol. Therefore, the analysis process must go through all the possibilities. For example, there are three symbolic inputs in Fig 6 which will result in six outputs. The process determines whether there is an input that makes the output of the two basic blocks the same. If there is, the two basic blocks are semantically equivalent. If not, the two basic blocks are not semantically equivalent.

#### 4.3.2. Basic block relationship analysis

Basic block semantic equivalence analysis can judge the functional changes inside the basic block. Some patches may not change the semantics of the basic block, but may change the judgment condition or jump path between basic blocks. Basic block semantic analysis cannot identify such patches. The jump relationships among basic blocks must be examined to analyze such patches. Every basic block is numbered based on their addresses when Angr generates the CFG and all paths of the CFG are traversed. The LCFG where the basic block is located is represented as a string, and the similarity of the LCFG can be obtained by comparing the similarity of strings.

The call instruction under *X*86 is *Call*, while the call instruction of *ARM* and *PPC* is *BL*, and the call instruction of *MIPS* is *jal* and *jalr*. We represent the call instruction as *Call* unified, and the string representation of the LCFG is shown as follow.

StrBB:node,call,…,call,Num_Neigh,Neigh1,…Neighn

And the content of the string representation is shown in Table 2.

**Table 2:** LCFG string representation.

Item	First item	Second item	Third item	Fourth item	Fifth item	Sixth item
String	Node	Call	Num_of_fathernode	FatherNode	Num_of_childnode	Childnode

The first item in the table is the index of the basic block, and the second item is the call instruction in the basic block. If there is no call instruction, the item is empty. The third item is the number of parent nodes of the first node, and the fourth item is the index of the parent nodes. The fifth item is the number of child nodes of the first node, and the sixth item is the index of the children nodes.

The LCFG has two-layer parent nodes and two-layer child nodes. For the first-layer node of the CFG, there are no parent nodes, while the second-layer node of the CFG has only single-layer parent nodes. The last layer of the CFG has no child nodes, and the next-to-last layer of the CFG has only single-layer child nodes. Each basic block and surrounding nodes can be represented as the following string:

$$StrBB_{n-2}-StrBB_{n-1}-StrBB_{n}-StrBB_{n+1}-StrBB_{n+2}$$

After analyzing a large number of firmware functions, it can be seen that the vulnerability function is relatively large and the number of basic blocks is relatively large, thus, in the actual analysis, the string representation of the LCFG is generally long. Because of its speed benefits, Google’s CityHash64 is used to find the 64-bit Hash value for the string representation. The similarity of two LCFG hashes is calculated by calculating the Jaccard distance:

$$\begin{array}{c} \begin{array}{c} Sim\left(H1,H2\right)=Jaccard\left(H1,H2\right)=\frac{\left|H1\cap H2\right|}{\left|H1\cup H2\right|}\\ =\frac{\left|H1\cap H2\right|}{\left|H1|+|H2|-|H1\cap H2\right|} \end{array} \end{array}$$ (3)

## 5. Implementation and evaluation

A prototype is implemented to verify the effectiveness of our method and to evaluate the prototype in three aspects: accuracy, efficiency, and utility. Real-world cases are used to verify the effectiveness of the prototype. The evaluation experiments were implemented with 4.3 GHz, 128 GB memory, 2 TB SSD, and a single GPU server.

### 5.1. Dataset and evaluation criteria

DataSet1: Sample DB. The data for this database comes from two sources: open-source databases commonly used in firmware and open-source firmware downloaded from github. The open-source databases we chose are *openssl*1.1.0*f* and *BusyBox*1.27.2. The libraries were compiled into three architectures (*ARM*, *MIPS*, *andX*86) by the compilers *gcc*6.4 and *clang*3.9 at the three optimization levels of *O*1, *O*2, and *O*3. The disassembler IDA7.0 was used to extract the function control flow features and Angr was used to extract the DDG features. The database contains 10 million functions. DataSet1 is mainly used to evaluate the accuracy of the method. Homologous functions under different architectures, compilers, and compile optimization are marked as 1, while non-homologous functions are marked as -1.

DataSet2: Firmware function DB. This dataset contains device firmware extracted by crawlers from the internet, including the firmware of routers, network attached storage (NAS), printers, among others. Binwalk was used to make a preliminary analysis of the firmware, reveal the basic information about the firmware processor architecture and operating system, and extract the file system from the firmware. An IDA plug-in is used to extract the CFG features and Angr is used to extract the DDG features. DataSet2 is mainly used to evaluate the efficiency of the method.

DataSet3: Vulnerability dataset DB. The data for this database comes from two sources: (1) the list of common vulnerabilities and exposures numbers of vulnerabilities maintained by the Mitre Corporation website (from which 50 vulnerability functions were obtained), and (2) firmware with bugs and firmware with patches. DataSet3 is used to evaluate the utility of the method.

Accuracy evaluation criteria: Using accuracy evaluation criteria in machine learning: ROC (receiver operating characteristic curve) and AUC (Area Under Curve). The AUC value is equivalent to the probability that a randomly chosen positive example is ranked higher than a randomly chosen negative example.

$$\begin{array}{c} AUC=\frac{\sum_{i\in positiveClass}rank_{i}-\frac{M(1+M)}{2}}{M\times N} \end{array}$$ (4)

Efficiency evaluation criteria: The time consumed by firmware function similarity analysis is divided into preparation time and search time. Preparation time includes firmware function disassembly time, feature extraction time and SimHash generation time, among which disassembly time can be excluded from the evaluation criteria because all methods use the same disassembly method. The search time is the data retrieval time.

$$t_{indexed}=t_{feature}+t_{SimHash}$$

$$t_{search}=t_{retrieval}$$

### 5.2. Accuracy

The accuracy evaluation of firmware function similarity analysis was implemented by using the data in DataSet1, and the accuracy evaluation was realized in two aspects: accuracy of the firmware function SimHash and comparison with the state-of-art methods.

#### 5.2.1. SimHash accuracy

**Fig 7:**
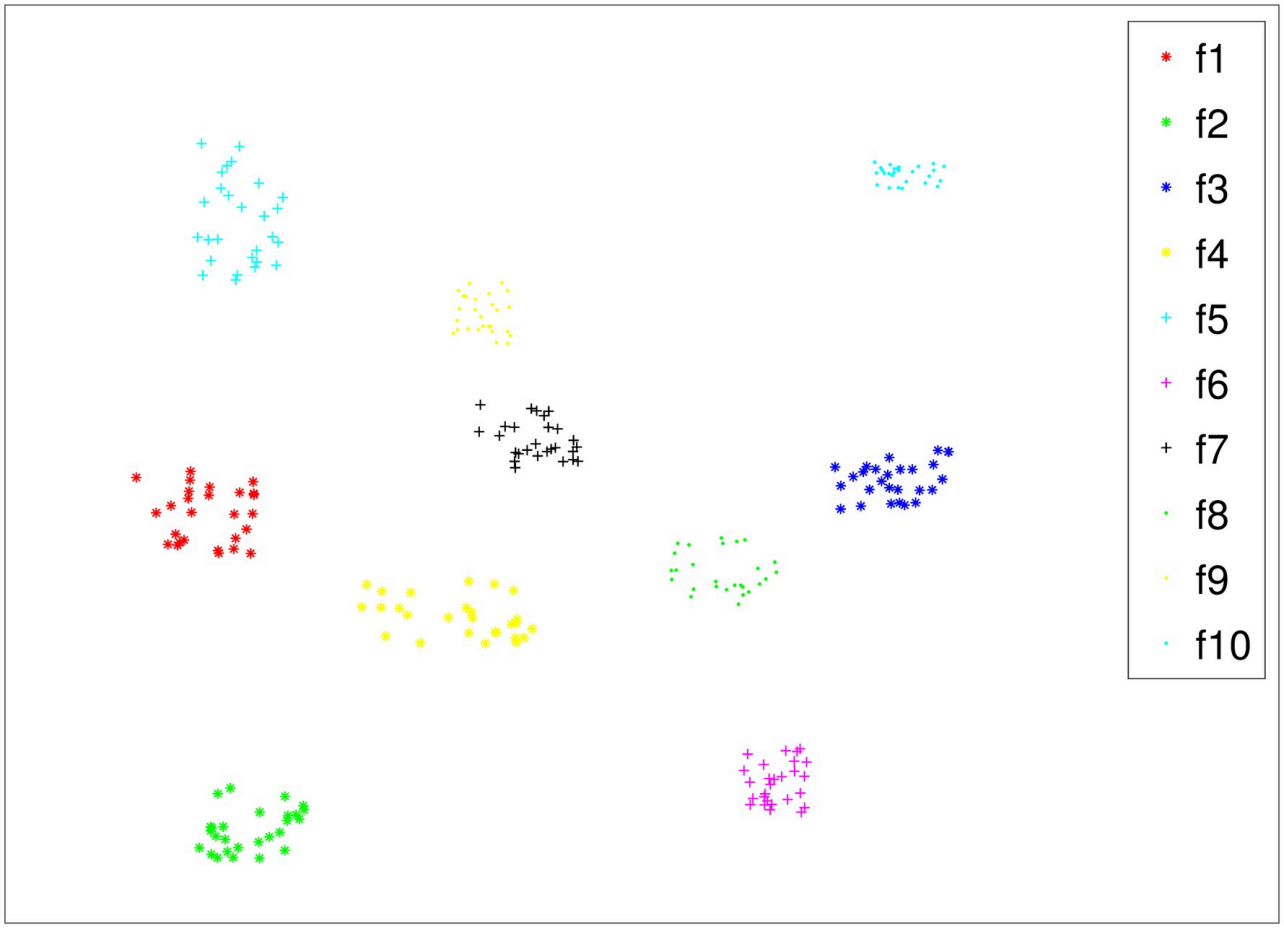
Visualizing function Simhash.

We convert the firmware function into SimHash using SimHash to implement firmware function similarity analysis. Therefore, the accuracy of SimHash directly determines the accuracy of firmware function similarity analysis. To provide a visual approach to evaluate the accuracy of SimHash, t-SNE [23] was used to map the function SimHash to a two-dimensional plane. Ten functions were randomly selected from DataSet1. Each function in DataSet1 has 18 representations (different compilers, different compilation optimizations, different processor architectures). Therefore, a function in DataSet1 has 17 homologous functions, and the SimHash of these 18 functions have high similarity, while the SimHash of non-homologous functions have low similarity. The clustering results of the ten functions on a two-dimensional plane are shown in Fig 7.

As shown in the figure, the same color homologous functions will be clustered together with a closer distance between them, while different functions have a greater distance.

#### 5.2.2. Comparison

The firmware function similarity analysis method in this study is divided into two phases: the first phase is to convert the firmware function into SimHash, which is used to implement firmware function similarity analysis; The second phase is to implement the fine-grained similarity analysis at the basic block level. The comparison evaluation is carried out in the first phase, first with Gemini [16] and StagedMethod [24], followed by comparison of the first phase and the second phase.

**Fig 8:**
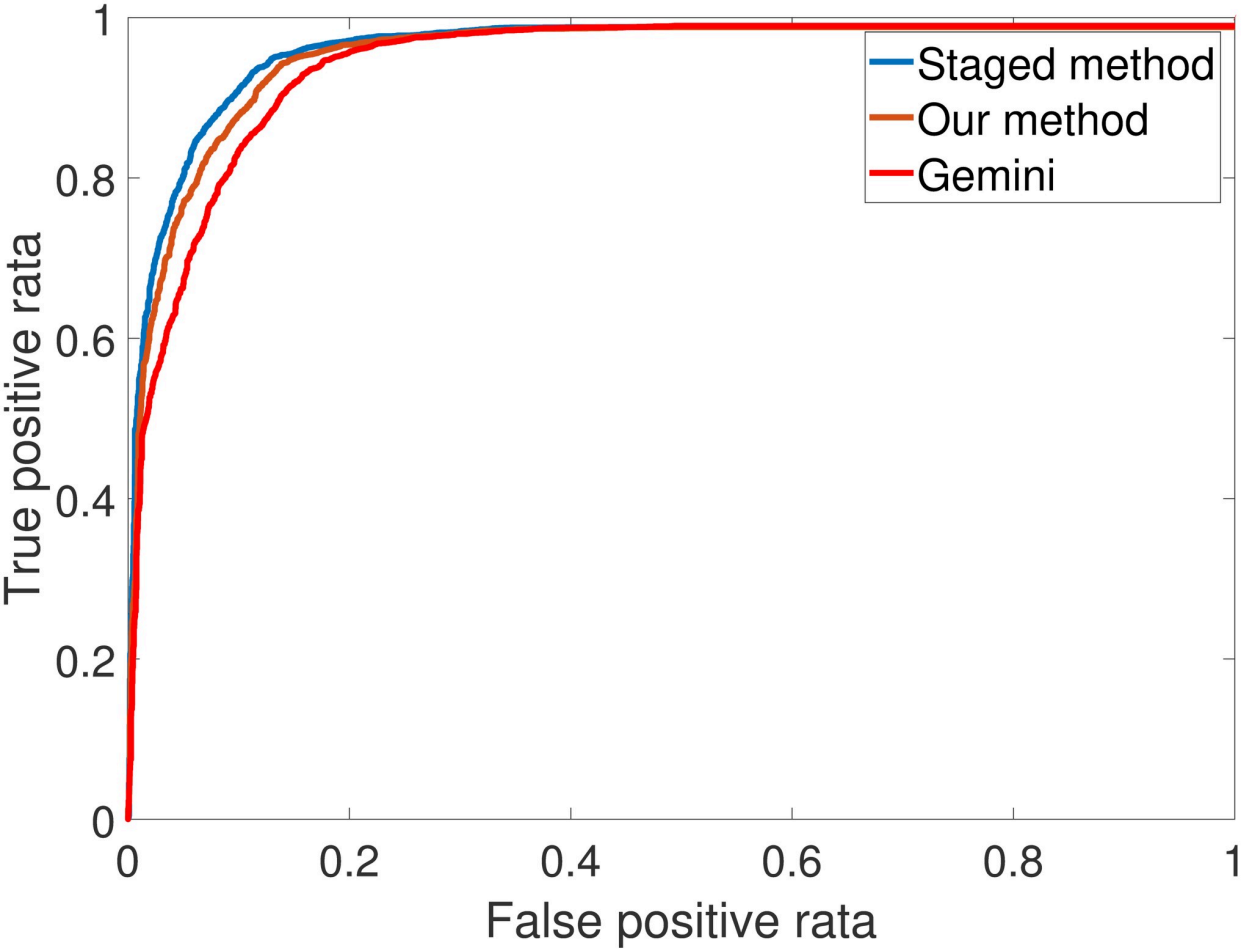
Visualizing function Simhash.

Fifty thousand pairs of functions were randomly selected from DataSet1, among which forty thousand pairs were homologous functions. Fig 8 illustrates the accuracy comparison results of our method, Gemini, and the StagedMethod. The experimental results demonstrate that the accuracy our method is higher than that of Gemini, but slightly lower than that of the Staged Method. Following the analysis, there are two possible reasons for this result: Firstly, the features we chose include control flow features and data flow features. Compared with Gemini, the feature variety of our method is richer and the number of features of our method is larger, so the accuracy rate is higher than that of Gemini; Secondly, our control flow features are all from the original features of the StagedMethod, and we did not consider the similarity of the function call flow graph which may make the accuracy of our method is slightly lower than StagedMethod.

We also compare the accuracy between first stage and second stage of our method, the result shows that the accuracy of the second stage is not improved greatly compared with the first stage. The reason may be that the similarity of basic blocks does not represent the similarity of functions. The basic block semantics may be equivalent, but functional semantics may be different. Moreover, the purpose of the second stage of our method is not to improve the accuracy, but rather to solve the problem of determining the firmware patch location without vulnerability function information.

### 5.3. Efficiency

Due to the large base of networking equipment and the large number of functions contained in the firmware, a large-scale firmware security inspection and detection method must be efficient. This section evaluates the efficiency of our method. The evaluation experiment is primarily aimed at the efficiency of the first phase of our method.

The time consumed by our method is divided into indexing time and search time, among which indexing time includes firmware function disassembly time, feature extraction time, and SimHash generation time. Search time is the retrieval time of all data in the dataset, and the search efficiency is different due to the different data storage and data retrieval approaches in the different methods. To evaluate the efficiency of the proposed method in the real environment, 100,000 firmware functions were randomly selected from DataSet2 to evaluate the efficiency of indexing and search, and to compare the efficiency with Gemini and StagedMethod.

#### 5.3.1. Indexing time evaluation

**Fig 9:**
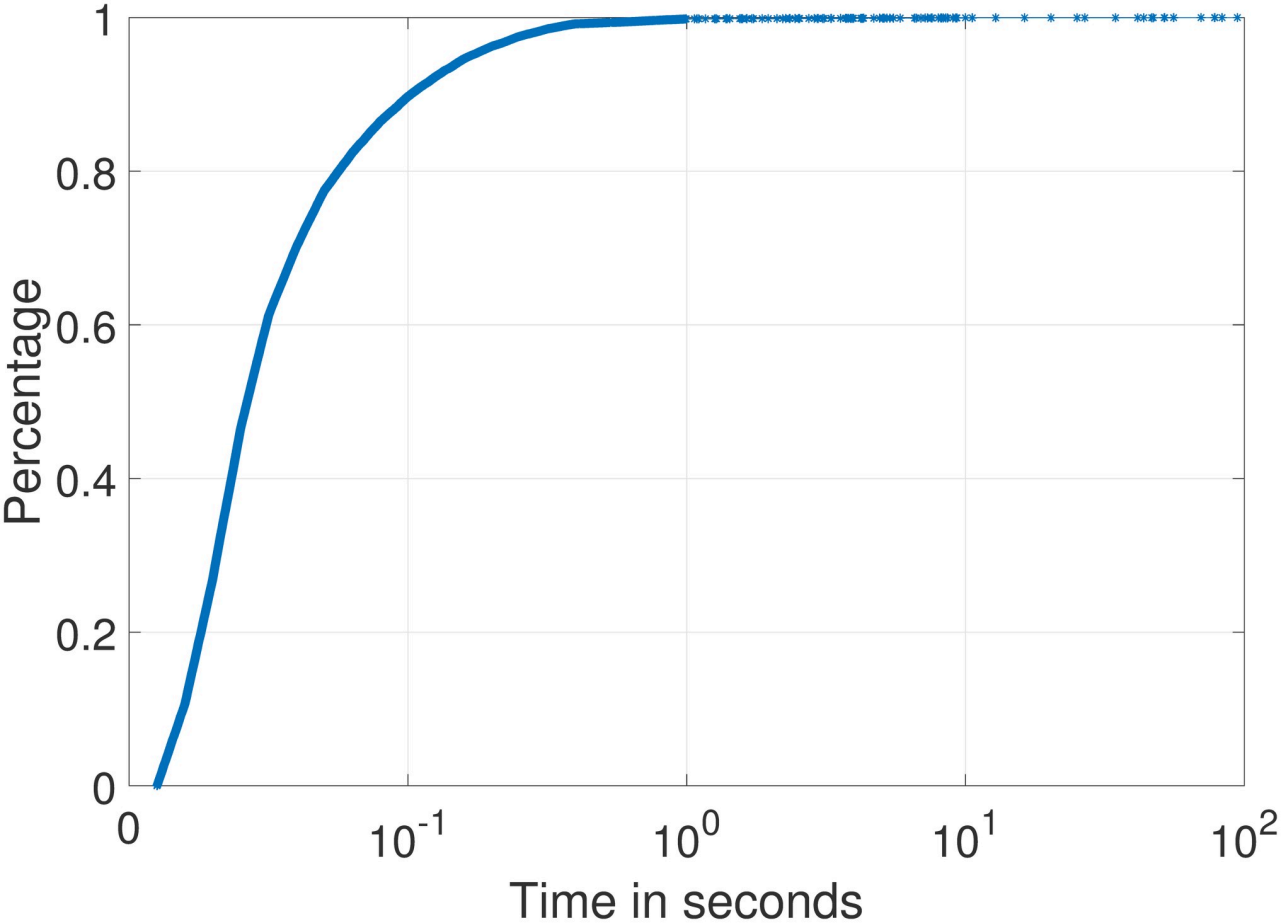
CDF graph of the indexing time.

Since the existing method is to disassemble the firmware function through the IDA plug-in, so is our method; therefore, the time required for disassembly of the firmware function can be ignored in the indexing time evaluation. The function indexing time of our method is the time to generate SimHash. Fig 9 is the cumulative distribution function (CDF) graph of the indexing time for the selected function. The indexing time of 90% of the functions is less than 0.1 s, while the time for other functions is more than 1 s, which is determined by the basic block number of functions. Experimental results show that the average indexing time of each function is 0.04s.

#### 5.3.2. Searching time evaluation

**Fig 10:**
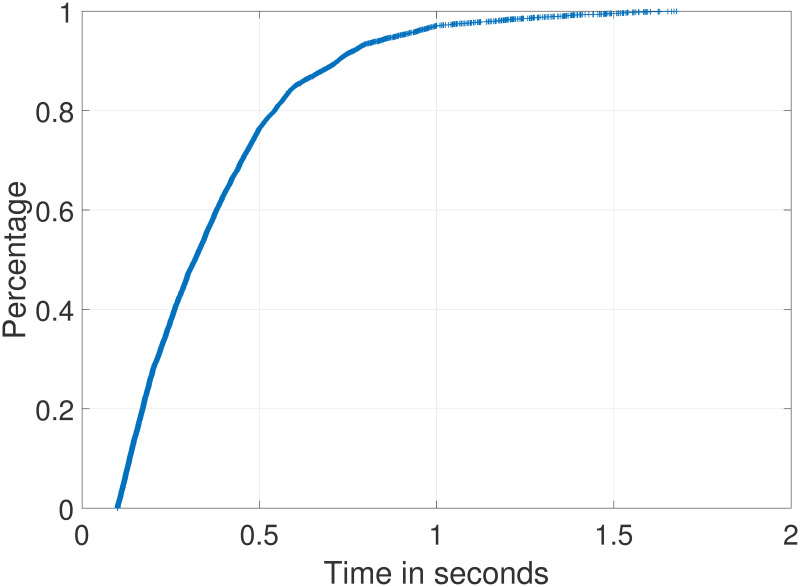
CDF graph of the search time.

Data indexing can be completed offline. After generating the indexed data, the firmware function security vulnerability detection can be completed through online search. The search time is a very important factor in evaluating the efficiency of the method. The experimental result is shown in Fig 10, completing a 100,000-function search within 2 s.

#### 5.3.3. Comparison

**Fig 11:**
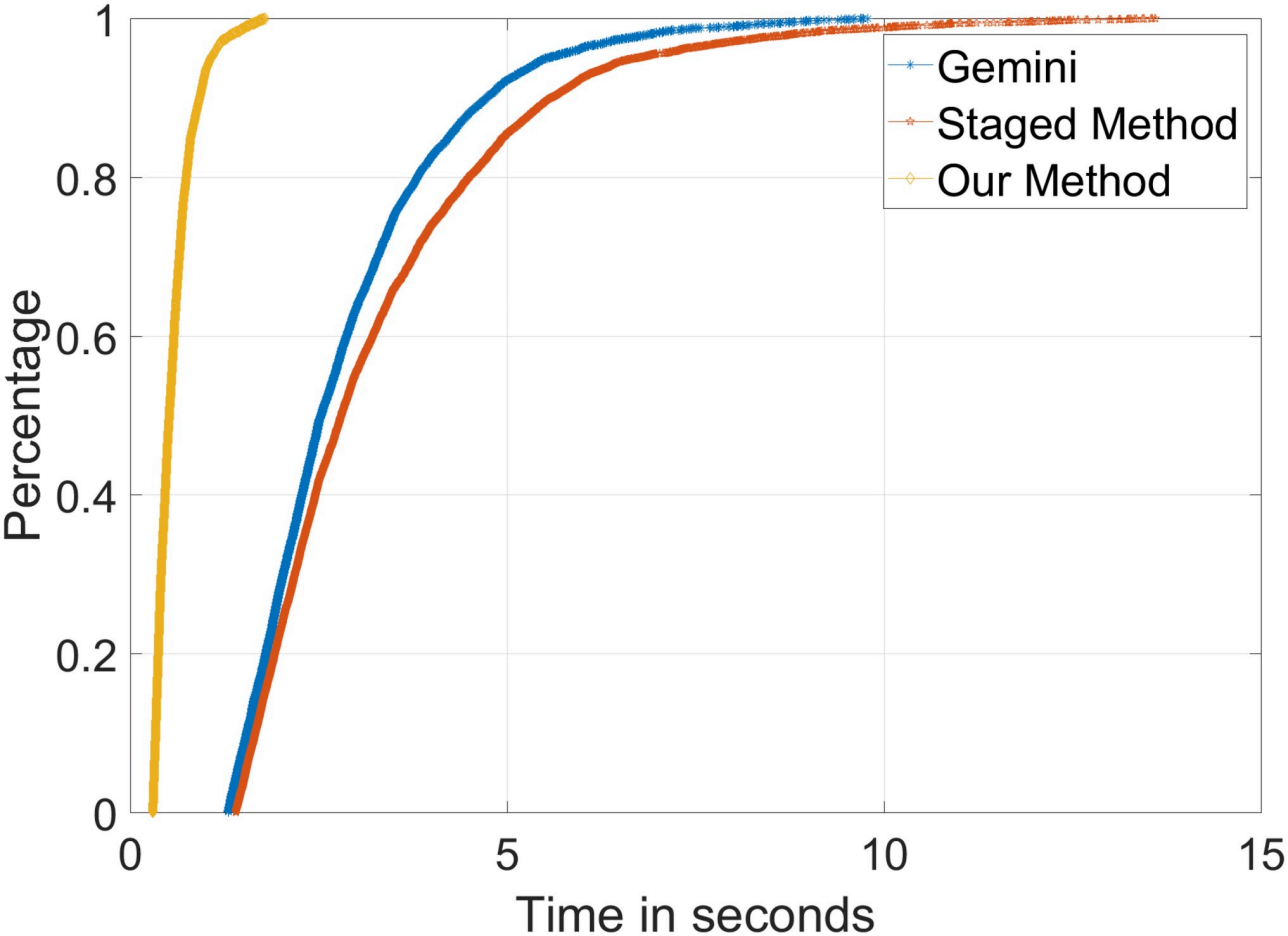
Efficiency comparison with Gemini and StagedMethod.

Comparing our method with Gemini and StagedMethod for efficiency, the result is shown in Fig 11. Because both Gemini and StagedMethod need to generate the function embedding by the neural network, the indexing time is relatively long, and the efficiency of the method in this study is higher than that of the other two methods.

### 5.4. Real-world case

To verify the effectiveness of the proposed method, we conducted experiments using a large quantity of real-world firmware (patched and unpatched). In this section a *TP* − *linkWR*940*N* router is selected as the example to verify the effectiveness of our method in function similarity analysis and basic block similarity analysis. The unpatched and patched firmware is downloaded from the network. Binwalk is used to analyze the firmware preliminarily, the firmware is not encrypted and the file system can be extracted by BinWalk. The binary file *httpd* is found in the firmware. In general, there is a high probability of there being a vulnerability in HTTP daemon, so we choose the *httpd* binary program to analyze.

#### 5.4.1. First phase analysis

The process of the first phase analysis is as follows:

1. IDA and Angr were used to disassemble the httpd and extract the function features
2. The SimHash of the disassembled firmware function was calculated
3. The SimHash similarities between patched and unpatched httpd were compared

The result of the comparison is shown in the Table 3.

**Table 3:** Result of first phase analysis.

	Similarity >98%	80%<Similarity<98%	50%<Similarity<80%	10%<Similarity<50%	No match	Total
Unpatched	3486	195	278	14	231	4204
Patched	3486	195	278	14	43	4016

From the analysis results, it can be seen that the similarities of more than 80% of the functions are more than 98%, and these functions belong to the same function. The functions that have similarity less than 98% are considered to be either patched or modified functions. Patches make small changes to a function in general, but there are a few cases with large changes. Unmatched functions can be discarded functions or new functions. To improve the analysis efficiency, further similarity analysis is only carried out for functions with similarity between 80% and 98%.

#### 5.4.2. Second phase analysis

**Fig 12:**
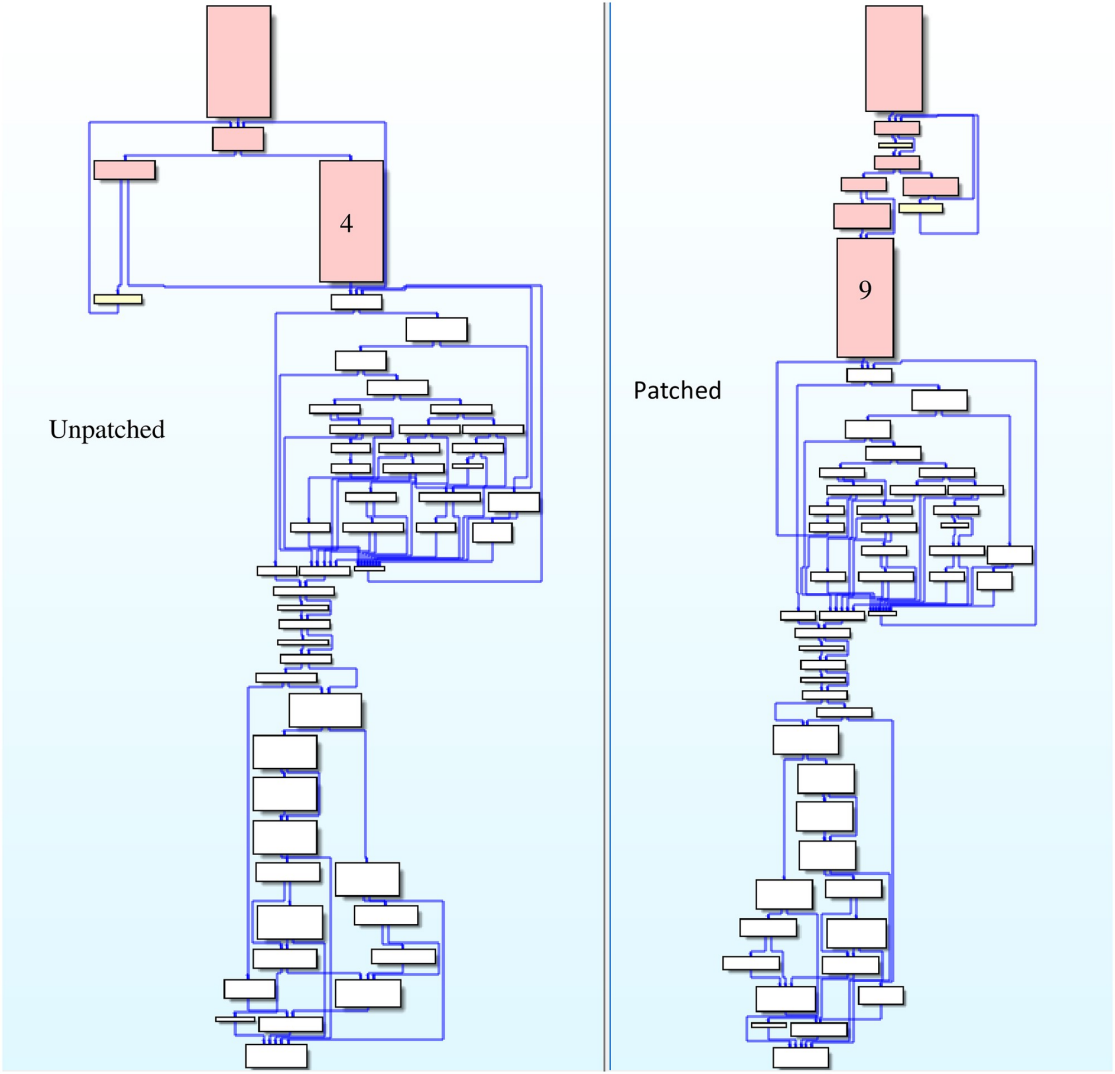
CFGs of function ipAddrDispose.

**Fig 13:**
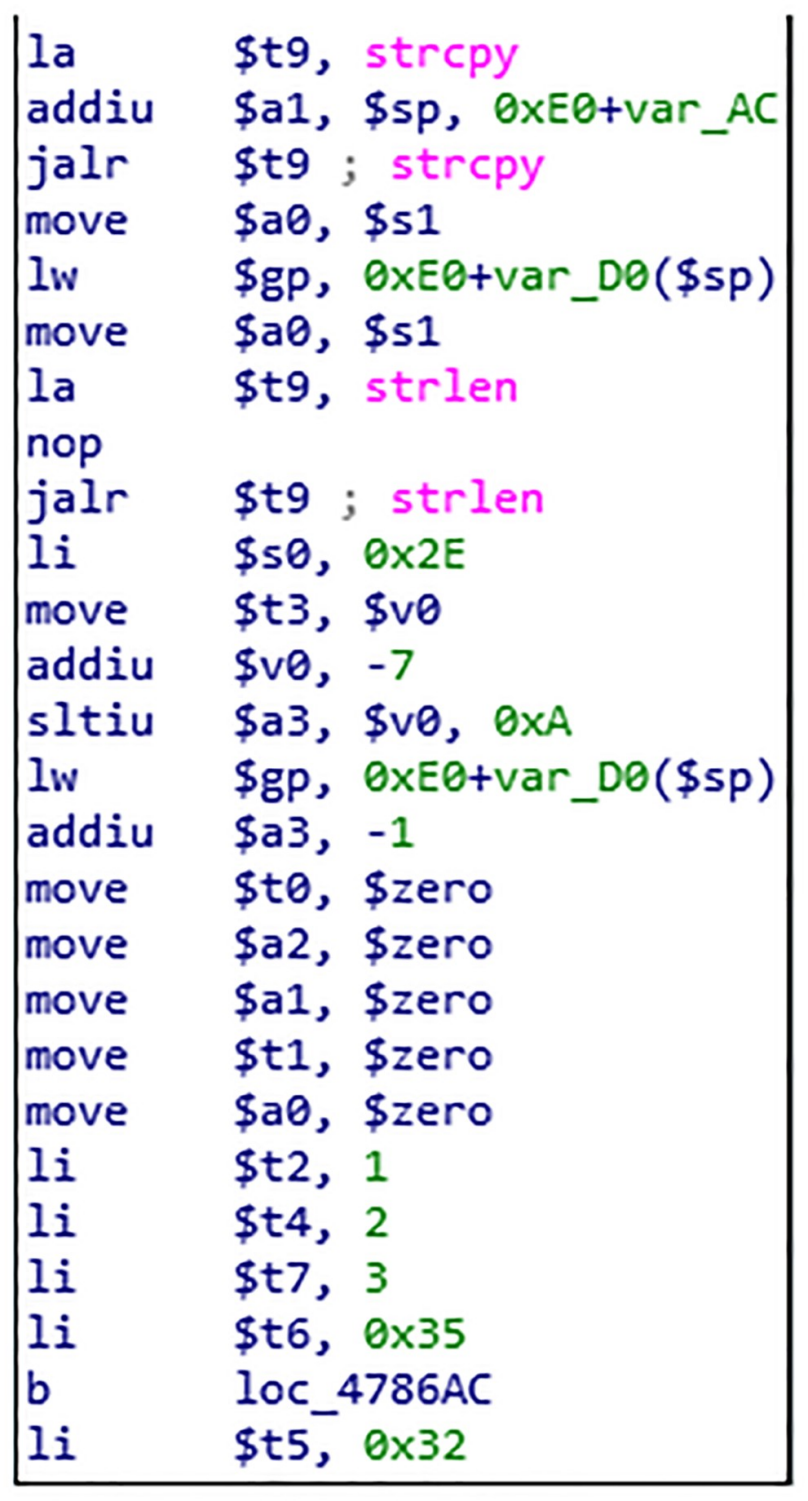
Unpatched firmware function BB.

**Fig 14:**
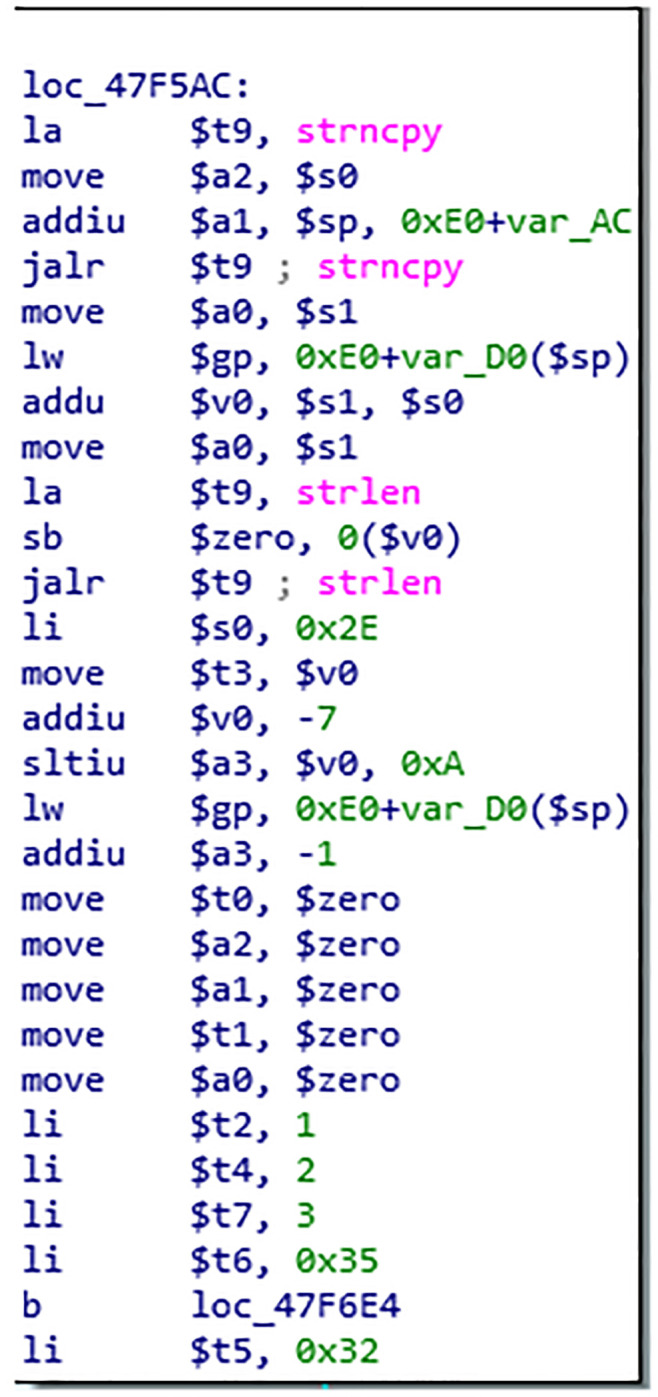
Patched firmware function BB.

The second phase implements the basic block-level similarity analysis based on the first stage analysis; some findings were made when analyzing the function *ipAddrDispose*. The CFG of the function *ipAddrDispose* is shown in Fig 12, where Fig 12(left) is the function of unpatched firmware and Fig 12(right) is the function of patched firmware. When analyzing the similarity of basic blocks of *ipAddrDispose*, the semantic of basic block 4 of Fig 12(left) is equal to the basic block 9 of Fig 12(right), but the location of the base block in the CFG changes. The basic block 4 and basic block 9 of the function *ipAddrDispose* are shown in Figs 13 and 14. The hash of the LCFG of the basic block was calculated, and then the distance similarity between the hash of different LCFGs of basic block 4 and basic block 9 was calculated. Their Jaccard similarity was 92%. Finally, after we made a manual confirmation, it was found that in the function of Fig 13, the *Strcpy* function, is called without length verification, which is a buffer overflow vulnerability, while in the function of Fig 14, the new function *Strncpy* is used and a length verification parameter is added. Although new parameter is added, the semantics of the two basic blocks are equivalent, and existing methods cannot find such vulnerabilities.

## 6. Discussion

Section 5 evaluates our method in three aspects: accuracy, efficiency, and utility, and compares the performance with the state-of-art methods: Gemini and StagedMethod. SimHash and basic block-level analyses are used in our method to overcome the shortcomings of incomplete features, high computing overhead, and poor extensibility. Experiment 5.2 confirms that the accuracy of our method is higher than that of Gemini. Experiment 5.3 confirms that the efficiency of our method is much higher than that of the Gemini and StagedMethod. Experiment 5.4 confirms the utility of our method.

Most of the existing firmware security analysis techniques require model training, which requires a large number of samples (similar and non-similar function pairs), and these samples are relatively expensive, while the numbers and types of embedded firmware in the IoT are very large; thus, it is difficult to embed all firmware code accurately. What’s more, when analyzing new firmware, it is sometimes necessary to retrain the model, requiring days or even weeks. This results in poor extensibility of existing methods. Our method does not need model training and can analyze the new firmware directly. Therefore, our method has good extensibility and is suitable for large-scale firmware function security analysis.

## 7. Related work

### 7.1. Traditional firmware code similarity analysis technique

Because the dynamic analysis of firmware is difficult, the traditional firmware code analysis technology is mainly static analysis, which can be divided into source analysis and non-source analysis. Source analysis methods include [25–29], among others, the firmware is closed-source mostly, and the source analysis of firmware is not applied in practice. There are many non-source firmware analysis researches. BinHunt [30] and iBinHunt [31] use symbolic execution and constraint solver to determine the semantic equivalence among binary programs. However, this method is too expensive to be applied to large-scale firmware analysis. Bindiff [32, 33] realized the similarity analysis of functions under different architectures by comparing CFGs of target function pairs; the efficiency of Bindiff is high, but the accuracy is low. Bingo [34] inline related library and user-defined functions to capture complete functional semantics, enabling cross-architectural code analysis. However, Bingo heavily depend on CFG which could be altered significantly because of different ISAs or variant compiling configurations. Multi-mh and multi-k-mh [7] realized function similarity comparison under different ISA architectures through fuzzy logic and graph matching, but this method has a high overhead. BinSequence [35] implements fuzzy function matching by comparing the longest common sub-sequence at basic block level and the neighbors of the basic block. Genius [8] extracts the features of CFG, converts them into high-dimensional vectors, and uses the clustering algorithm to obtain a codebook, which embeds the target function. However, the performance of generating codebook is expensive. Firmup [36] performs firmware function CFG matching directly and calculates the semantic similarity of functions, which will have high accuracy but high performance overhead. David et al. [20] and [37] implement function similarity analysis by data stream slicing.

### 7.2. New firmware code similarity analysis technology

Machine learning has been increasingly applied in the field of code analysis, and has achieved good effects [23, 38–40]. Gemini designs a neural network which is used to generate function embedding, which improves the efficiency. Compared to Gemini, the StagedMethod [17] increases the function of local call flow graph similarity analysis phase, which improves the efficiency and accuracy. Instruction2vec [41] converts functional instructions into vectors for similarity analysis. ASM2vec [42] converts the function into numerical vectors. Both Instruction2vec and ASM2vec are only applicable to uniprocessor architectures, however, their implementation method has guiding value for cross-architectures and cross-OS code similarity analysis. API2Vec [43] converts APIs into vectors, which is suitable for source code-level similarity analysis. DeepRepair [44], NP-CNN [24] and LS-CNN [45] are also source code-level similarity analysis methods. α_Diff_ [46] designed a deep neural network (DNN) to learn function features from raw bytes to realize the cross-version binary similarity analysis. SPAIN [47] is a scalable binary-level patch analysis framework, which can automatically identify security patches and summarize patch patterns and their corresponding vulnerability patterns. Jian Gao et al. present Vulseeker [48], a semantic learning based vulnerability seeker for cross-platform binary. Yue Duan present DeepBinDiff [49],an unsupervised program-wide code representation learning technique.

## 8. Conclusions

In this study, we have proposed a high-efficiency similarity analysis approach for firmware code. The approach could determine the similarity of firmware functions by calculating the similarity of the function SimHash. Due to the high computational efficiency of SimHash, our method can implement large-scale security inspection of firmware functions efficiently. By analyzing the semantic equivalence among the basic blocks and the similarity of the LCFG, the location of a firmware patch can be obtained without detailed information of the vulnerability function. Compared to the existing firmware similarity analysis methods, our method improves the efficiency, ensures the accuracy, and solves the new problem of locating the firmware patch. We designed a prototype and compared it with state-of-the-art methods. The experimental results show that the efficiency of our method is much higher than Gemini and StagedMethod, and the accuracy is higher than Gemini. The experiment involving the TP-link WR940N router proves that our method can obtain the location of a firmware patch without vulnerability function information. Moreover, our method does not need model training and can analyze the unknown firmware directly.

In our future work, we will further study the basic block semantic similarity, including the situation of function inlining, function structure modified, etc., and evaluate the efficiency of basic block similarity analysis. We can further improve upon the accuracy and efficiency of our method. Our control flow features are all from the original features of the StagedMethod without filtering; some features may affect the accuracy of SimHash. More feature is not always better, some of them are low important or redundant. Features of low importance refer to those that contribute less to improving the accuracy of code similarity analysis, mainly including instruction distribution features and CFG branch structure features. The reason for the low importance of these features is that the instruction distribution of different functions does not differ much. Redundant features are features that have the same attributes and can be derived from each other. For example, the number of basic blocks and the number of function transfer instructions are both important, but the number of function transfer instructions can be inferred from the number of basic blocks, so one of the features can be discarded. Therefore, the next step is to evaluate and filter the features, to remove duplicate features and add useful features. We will extract firmware function features in new ways, for example, machine learning. The overhead of locating firmware patches by basic block-level analysis is very high. Thus, the next research step would be to explore new methods to locate firmware patches.
